# Case report: Primary subcutaneous Rosai-Dorfman-Destombes of the scalp with intra-cranial involvement: diagnosis and treatment of a rare case with literature review

**DOI:** 10.3389/fneur.2025.1557385

**Published:** 2025-02-19

**Authors:** Qin Zhenwei, Tang Beiyan, Chen Ding, Liang Qiang, Dong Qiang, Zhai Huixia, Kang Wei, Zhao Xianjun, Pan Yawen

**Affiliations:** ^1^Department of Neurosurgery, The Second Hospital & Clinical Medical School, Lanzhou University, Lanzhou, China; ^2^The Second Hospital & Clinical Medical School, Lanzhou University, Lanzhou, China; ^3^Gansu Province Hospital Rehabilitation Center, Lanzhou, China; ^4^Lanzhou New District First People’s Hospital, Lanzhou, China; ^5^Key Laboratory of Neurology in Gansu Province, Lanzhou, China

**Keywords:** Rosai-Dorfman-Destombes disease, histiocytic proliferation, imaging evaluation, neuronavigation, surgical treatment

## Abstract

Rosai-Dorfman-Destombes disease (RDD) is a rare histiocytic proliferative disorder, with primary scalp and intracranial involvement being particularly uncommon. The imaging features and clinical manifestations of RDD often overlap with other intracranial lesions, such as meningiomas, leading to misdiagnosis. This study presents a case of primary scalp and intracranial RDD, with a comprehensive analysis of its imaging, pathological, and intraoperative findings, alongside a review of the literature on central nervous system (CNS) RDD and its diagnostic and therapeutic advancements. Preoperative CT and MRI scans clearly depicted characteristic changes in the scalp and intracranial lesions. However, the preoperative assessment failed to fully recognize abnormalities in the skull base, leading to an incomplete initial understanding. During surgery, the dura mater and obstructed superior sagittal sinus were resected extensively, and the pathology confirmed RDD. Postoperative recovery was smooth, and no recurrence was observed during follow-up. This case emphasizes the importance of detailed imaging in the diagnosis and treatment of RDD, combining preoperative evaluation with intraoperative observations to reduce the risk of misdiagnosis and recurrence. Furthermore, the exploration of individualized treatment strategies and targeted therapies plays a crucial role in managing complex cases. This study offers valuable experience for the diagnosis and treatment of similar rare cases.

## 1 Introduction

Rosai-Dorfman-Destombes disease (RDD) is a rare non-Langerhans cell histiocytosis, with an incidence of approximately 1 in 200,000. The disease was first reported by Destombes (1) in 1965, and subsequently, in 1969, Rosai and Dorfman (2) conducted a detailed study of 34 patients and characterized the disease as “sinus histiocytosis with massive lymphadenopathy.” In 1987, RDD was classified as a non-Langerhans cell histiocytosis, characterized by extensive accumulation and infiltration of pale histiocytes. In the revised classification of histiocytosis in 2016, cutaneous RDD was categorized as Group “C,” while familial, classic, extranodal, tumor-associated, and immune-related RDD were classified under Group “R” (3). In 2023, the fifth edition of the World Health Organization (WHO) classification of hematologic and lymphoid tissues (lymphoid tumors) included RDD under the category of histiocytic/macrophage tumors, recognizing it as a true neoplastic disorder (4).

The typical clinical presentation of RDD includes painless enlargement of one or more lymph nodes, often accompanied by fever, elevated neutrophil count, accelerated erythrocyte sedimentation rate, and hyperglobulinemia. Although RDD primarily affects the lymphatic system, there have been reports of extranodal involvement, affecting sites such as the skin, nasal cavity, orbit, and even multiple organs such as the salivary glands, spleen, pancreas, and testes (5, 6). Extranodal RDD accounts for approximately 43% of all RDD cases, while primary central nervous system (CNS) involvement is relatively rare, comprising about 5% of all RDD cases. The male-to-female ratio of the disease is approximately 1.8:1, with a higher incidence observed in males aged 40–50 years (7, 8). This article reports a case of primary scalp and intracranial RDD diagnosed and treated at the Second Hospital of Lanzhou University, and discusses it in conjunction with relevant literature, aiming to provide a reference for the diagnosis and treatment of similar cases.

## 2 Clinical data

**Figure 1:**
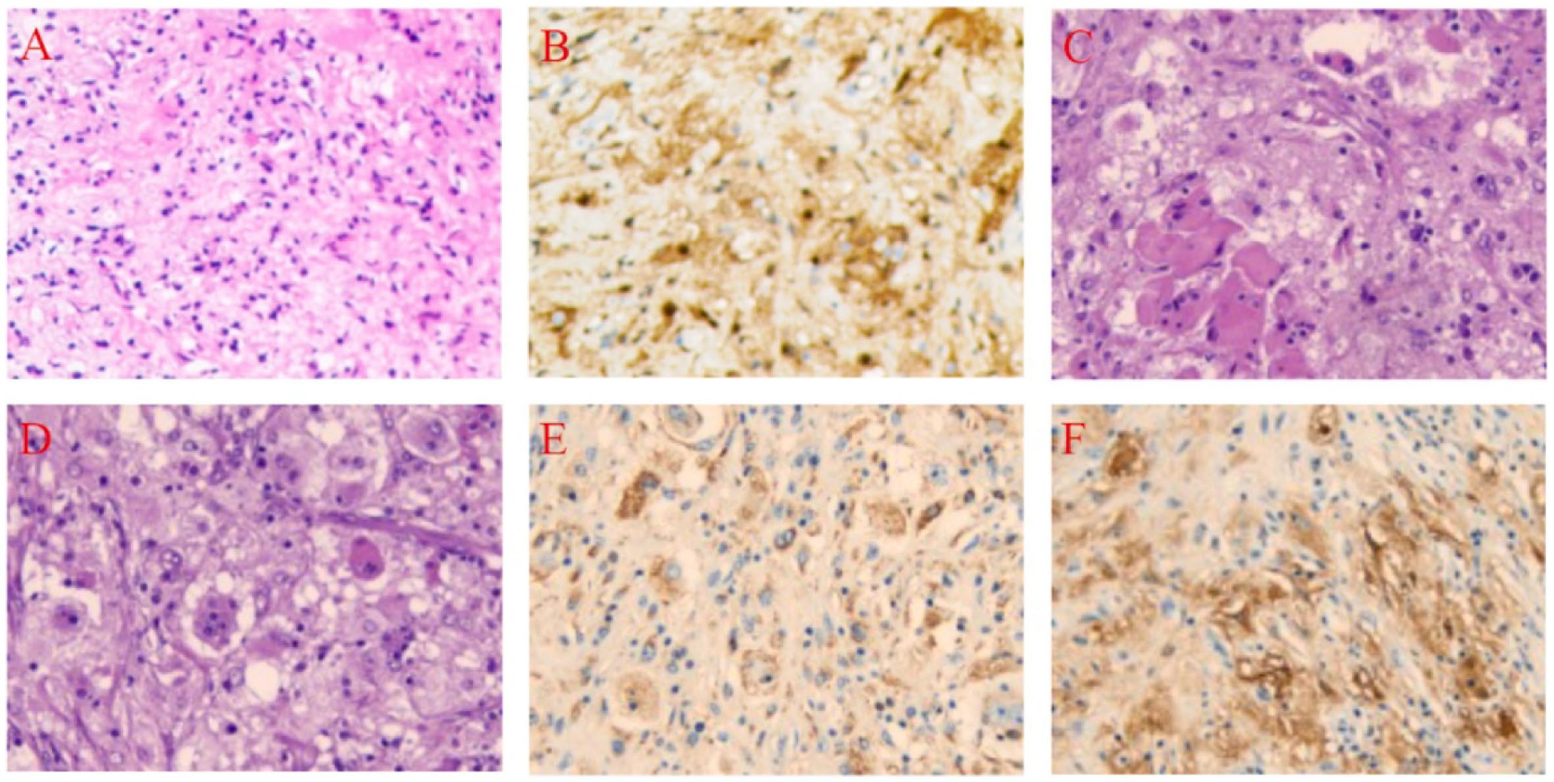
Pathological Findings Before and After Treatment in a 48-Year-Old Female with Scalp-Intracranial RDD. **(A)** Core biopsy, Hematoxylin and Eosin (HE), 10 × 20. **(B)** Core biopsy, S-100 positive, Immunohistochemistry (IHC), 10 × 20. **(C)** Histiocytes and perivascular plasma cell infiltration, HE, 10 × 20. **(D)** Lymphocytes extending into the histiocyte cytoplasm, HE, 10 × 20. **(E)** CD68 positive, IHC, 10 × 20. **(F)** S-100 positive, IHC, 10 × 20.

The patient is a 48-year-old female who was admitted to the hospital on November 18, 2024, due to a “painless mass on the scalp for over a year.” On November 7, 2024, the patient underwent a fine needle aspiration biopsy of the scalp mass at the First People’s Hospital of Lanzhou New Area. The postoperative pathological result indicated RDD (Figures 1A,B). Upon admission, physical examination revealed a mass beneath the scalp on the vertex of the head, which was oval-shaped, with a maximum diameter of approximately 9 cm. On palpation, the mass was firm, non-fluctuant, without tenderness or arterial pulsation. The overlying scalp was slightly erythematous, with local protrusion, and there was no ulceration (Table 1).

**Table 1:** Timeline of care of the patient.

Date	Event
2023–11	Symptom Onset: Painless scalp mass
2024-11-07	Initial Biopsy: Scalp RDD diagnosis confirmed
2024-11-18	Hospital Admission
2024-11-19	Preoperative Imaging: MRI and CT
2024-11-21	Angiography: Superior sagittal sinus involvement detected
2024-11-22	Surgery: Tumor resection and dura repair
2024-11-28	Postoperative Management: Subcutaneous fluid accumulation addressed
2024-01-15	Follow-up: No recurrence observed

### 2.1 Preoperative initial assessment

Preoperative hematological and biochemical analyses (Table 2) revealed key abnormalities associated with immune dysregulation and metabolic activity, including decreased lymphocyte counts, reduced complement C1q levels, and elevated lactate dehydrogenase and alpha-hydroxybutyrate dehydrogenase levels.

**Table 2:** Key hematological and biochemical findings relevant to the diagnosis of the patient.

Indicator	Value	Reference range	Clinical relevance
White Blood Cell Count (WBC)	4.34 ×10^9/L	4.00–10.00 ×10^9/L	Normal
Neutrophil Ratio (NE%)	73.10%	40–70%	Elevated
Lymphocyte Ratio (LY%)	17.50% ↓	20–40%	Decreased
Monocyte Ratio (MO%)	7.60%	2–8%	Normal
Eosinophil Ratio (EO%)	1.60%	1–4%	Normal
Basophil Ratio (BA%)	0.20%	<1%	Normal
Neutrophil Count (NE#)	3.17 ×10^9/L	2.00–7.00 ×10^9/L	Normal
Lymphocyte Count (LY#)	0.76 ×10^9/L ↓	1.00–4.00 ×10^9/L	Decreased
Monocyte Count (MO#)	0.33 ×10^9/L	0.10–0.60 ×10^9/L	Normal
Eosinophil Count (EO#)	0.07 ×10^9/L	0.02–0.50 ×10^9/L	Normal
Basophil Count (BA#)	0.01 ×10^9/L	0.01–0.10 ×10^9/L	Normal
Hemoglobin (HGB)	124 g/L	120–160 g/L	Normal
Mean Corpuscular Hemoglobin Concentration (MCHC)	311 g/L ↓	320–360 g/L	Decreased
Complement C1q	118.0 mg/L ↓	120–180 mg/L	Decreased
Lactate Dehydrogenase (LDH)	332 U/L ↑	140–280 U/L	Elevated
Alpha-Hydroxybutyrate Dehydrogenase (HBDH)	309 U/L ↑	72–182 U/L	Elevated

**Figure 2:**
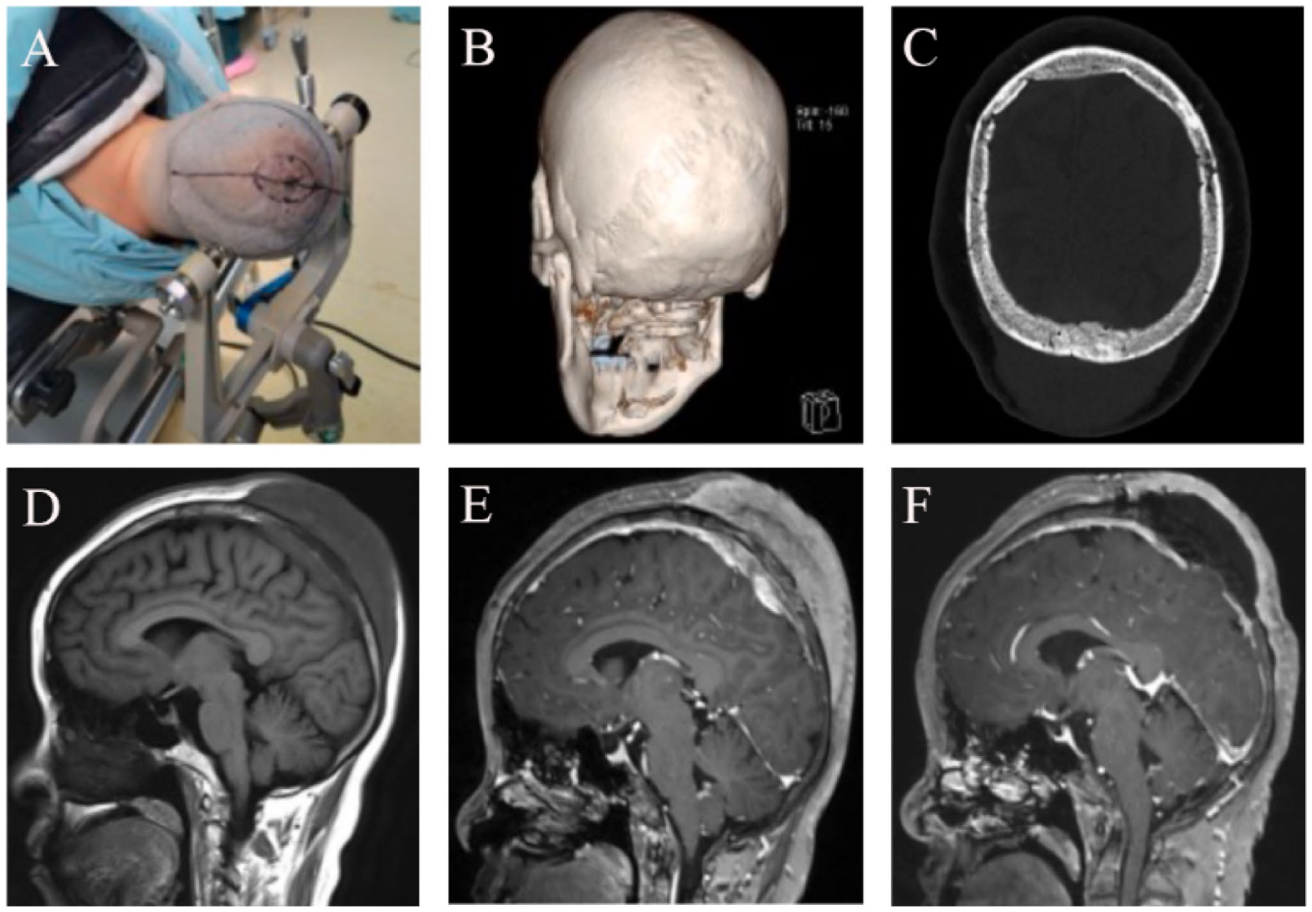
Imaging Findings Before and After Treatment in a 48-Year-Old Female with Scalp-Intracranial RDD. **(A)** Neuronavigation was used to mark the intracranial lesion site, and a wide-based flap was designed around the subcutaneous lesion. **(B)** Preoperative 3D CT showed no obvious erosion or destruction of the sagittal suture or the outer table of the skull. **(C)** Postoperative CT showed patchy high-density areas within the bone plate, suggestive of tumor-like changes in the bone. **(D)** Preoperative head MRI T1 sequence showed the bone plate appearing hypointense, with both intracranial and subcutaneous tumors displaying homogeneous low signal. **(E)** Preoperative enhanced MRI showed enhancement of the bone plate, intracranial tumor, and subcutaneous tumor, all presenting with high signal. **(F)** Postoperative follow-up enhanced MRI revealed subcutaneous fluid accumulation and a hypointense bone plate, suggesting ischemia due to the loss of blood supply in the bone flap.

Three-dimensional CT of the head performed at our hospital showed that the inner and outer tables of the skull were intact and smooth, with no significant bone defects. Head MRI revealed a subcutaneous mass on the vertex, measuring approximately 9 × 7.5 × 5 cm. The inner and outer tables of the skull were intact, and an intracranial mass involving the superior sagittal sinus was present, measuring about 5.5 × 4 × 2 cm (Figure 2). On November 21, 2024, cerebral angiography revealed near-complete occlusion of the superior sagittal sinus due to tumor infiltration, with extensive collateral circulation formation. The angiogram showed venous reflux through the anterior orbital floor and superior and inferior communicating veins into the cavernous sinus and superior petrosal sinus, eventually returning to the sigmoid sinus, with significant collateral circulation formed around the bilateral sagittal sinuses.

### 2.2 Surgical method

On November 22, 2024, under neuronavigation guidance, the intracranial lesion site was marked, and a wide-based “n”-shaped scalp flap was designed around the subcutaneous lesion. The scalp was incised in layers, revealing a subcutaneous tumor infiltrating the dermis, fat layer, and galea aponeurotica. The tumor was yellowish-white, firm in texture, moderately vascularized, and well-demarcated. Using a sharp blade, the lesion was resected in sections. Due to the thin epidermal layer and the potential for extensive scalp defects with complete removal of the subcutaneous tumor, resection was limited to the relatively normal subcutaneous tissue. Deeper exploration revealed no apparent defects or signs of invasion in the periosteum. Upon retracting the scalp flap, the periosteum was tightly adherent to the skull at the sagittal suture, but the boundary between the periosteum and the outer table of the skull was otherwise clear. The outer table of the skull across the surgical field appeared smooth, intact, and exhibited no abnormal color or texture.

A cross-midline cranial bone flap measuring approximately 7 × 5 cm was created under neuronavigation. Upon re-examining the inner table of the skull, no significant tumor invasion or abnormalities were observed. The dura mater was markedly thickened, and the intracranial tumor shared a similar appearance and consistency with the subcutaneous lesion. The tumor was irregular in shape, primarily located on the left side of the superior sagittal sinus, and exhibited a richer blood supply than the subcutaneous lesion. The sinus cavity was almost entirely obliterated. The tumor, along with the involved dura mater and superior sagittal sinus, was completely resected. Intraoperative bleeding was minimal. After repairing the dural defect, the cranial bone flap was repositioned and secured.

Postoperatively, the extensive flap area resulted in subcutaneous fluid accumulation, which was managed with fluid aspiration and compression dressing for symptomatic relief.

### 2.3 Pathological results

Histopathological examination revealed a large number of abnormally proliferating histiocytes with abundant cytoplasm. Lymphocytes were seen infiltrating the cytoplasm, with stromal hyaline degeneration and multinucleated cells present. Immunohistochemical staining results were as follows: tumor cells were positive for Vimentin (+), S-100 (+), CD68 (+), CD163 (+), Cyclin D1 (+); negative for CD1α (−), Langerin (−), GFAP (−), Olig-2 (−), EMA (−), Syn (−), CKp (−), p53 (wild-type), ATRX (+), SSTR2 (−), LCA (−), CD38 (plasma cell positive), CD3 (T cell positive), CD20 (B cell positive). The Ki67 labeling index was 3% (Figures 1C–F).

## 3 Literature review

RDD is a rare and complex disorder, and much of the research on its pathogenesis, clinical features, and treatment strategies is still evolving. Below is a review of recent studies that provide insights into various aspects of the disease.

### 3.1 Etiology and pathogenesis

The exact etiology and pathogenesis of RDD remain unclear. It is currently believed that the disease may be related to multiple factors, including infections, immune abnormalities, and genetic factors. Among the infectious factors, viruses (such as Epstein–Barr virus and human herpesvirus 6) and bacteria (such as Brucella) have been suggested as potential triggers, although there is no conclusive evidence to support these associations (9, 10). Immune abnormalities are considered a key mechanism in the pathogenesis of RDD. Studies have shown an imbalance in the proportion of CD4+ and CD8+ T cells in peripheral blood, which may lead to the abnormal activation and proliferation of histiocytes (9, 11). Additionally, gene mutations such as KRAS and MAP2K1, as well as defects in the Fas/FasL signaling pathway, have been detected in some cases of RDD, suggesting that genetic factors may play a role in disease development (12, 13). Overall, RDD is likely the result of a multifactorial interplay, involving immune responses triggered by infections, genetic susceptibility, and the influence of the inflammatory microenvironment. Further research is needed to better understand the pathogenesis of RDD and to develop targeted therapeutic strategies.

### 3.2 Clinical features and diagnosis

RDD is a rare histiocytic proliferative disorder with a diverse range of clinical presentations. Patients’ chief complaints vary widely, from asymptomatic skin nodules to thrombocytopenia. The hallmark feature of RDD is painless lymphadenopathy, most commonly involving the cervical lymph nodes, which account for 80–90% of cases. In addition, about 40% of patients have extranodal involvement, with affected sites including the skin, nasal cavity and paranasal sinuses, bones, orbits, genitourinary system, and central nervous system (CNS) (14). When the skin is involved, it manifests as polymorphic rashes or nodules. Skeletal lesions typically present as osteolytic foci or bone destruction (15, 16). CNS involvement may present with symptoms such as headaches, seizures, or focal neurological deficits (17).

Imaging findings typically show well-defined, contrast-enhancing soft tissue lesions at the affected sites. Cranial and brain CT scans of RDD often reveal homogeneous high-density or isodense soft tissue masses, sometimes accompanied by bone destruction or abnormalities in the skull plate. MRI findings typically show low signal on T1, iso- or high signal on T2, and uniform or heterogeneous enhancement after contrast administration. Intracranial lesions often involve the dura mater or the skull and may be confused with meningiomas (18).

Systemic symptoms in RDD are rare, with only a small proportion of patients presenting with fever, weight loss, or night sweats. Laboratory findings may show mild anemia, accelerated erythrocyte sedimentation rate (ESR), or hypergammaglobulinemia, but these are not specific indicators. The course of RDD is typically slow, and while some cases may resolve spontaneously, those involving vital organs may lead to severe complications. Therefore, the clinical presentation of RDD requires comprehensive assessment through pathological and immunohistochemical analysis to confirm the diagnosis and evaluate the prognosis.

### 3.3 Pathological features

RDD is a rare histiocytic proliferative disorder that can pose a challenge to clinical and laboratory diagnosis. The characteristic pathological features are of significant diagnostic importance (19, 20). Pathologically, RDD is characterized by alternating light-staining areas composed of histiocytes and dark-staining areas consisting of inflammatory cells such as plasma cells and lymphocytes, forming diffuse or nodular lesions. In some cases, histiocytes exhibit “emperipolesis,” where lymphocytes are phagocytosed by the histiocytes (21). Under hematoxylin and eosin (HE) staining, the typical appearance shows large histiocytes phagocytizing multiple smaller lymphocytes. Additionally, various forms of histiocytes and lymphocytes present in the lesion often exhibit an “alternating light and dark” irregular distribution pattern.

Although the classic presentation of RDD is bilateral cervical lymphadenopathy, it can also present with heterogeneous manifestations at various extranodal sites, including the skin, bones, kidneys, pancreas, central nervous system, and other organs. Due to the difficulty in identifying the characteristic histiocytes, the diagnosis of RDD can be complicated. Therefore, it is essential to use immunohistochemical staining for further differentiation. RDD histiocytes typically express CD68 (KP-1), CD163, CD4, CD11c, Cyclin D1, and Octamer-binding transcription factor 2 (Oct-2) (9, 22). Among these, S-100 positivity has greater diagnostic specificity, as some studies have reported CD86 being negative and CD1a weakly positive in RDD cases (23).

In non-specific inflammatory backgrounds, histiocytes are often sparsely distributed and difficult to locate, accompanied by intense infiltration of inflammatory cells. This can lead to confusion with other diseases, such as Langerhans cell histiocytosis, plasma cell granulomas, and meningiomas, during pathological examination. Therefore, the application of immunohistochemical staining is crucial for the differential diagnosis of RDD. Markers such as CD68 (KP-1), S100, and CD1a should be assessed for accurate diagnosis.

### 3.4 Differential diagnosis

RDD has a wide spectrum of clinical manifestations, and when it involves the lymph nodes or meninges, it can easily be confused with several other diseases. For accurate diagnosis and formulation of an appropriate treatment strategy, it is important to differentiate RDD from other diseases that share similar clinical or radiological features. These diseases include Langerhans cell histiocytosis (LCH), Erdheim-Chester disease (ECD), meningiomas, meningeal lymphoma, and idiopathic hypertrophic pachymeningitis. By comparing the pathological features, immunohistochemical staining, radiological findings, and clinical course of these diseases, the diagnostic characteristics of RDD can be more clearly defined and other possible diagnoses effectively ruled out. Below are the main points for differentiating these diseases:

1. Langerhans Cell Histiocytosis (LCH): LCH is primarily composed of Langerhans cells, which have lobulated nuclei and characteristic cytoplasm. It is often associated with bone involvement, visceral organ involvement, and skin lesions. Immunohistochemically, LCH is positive for CD1a and S100 (21). In contrast, RDD predominantly involves macrophages, which show phagocytosis of lymphocytes or red blood cells. Immunohistochemically, RDD is CD1a-negative, with positive staining for S100 and CD68. Clinically, LCH is more common in children and involves multiple systems, often with a poor prognosis, while RDD is more common in young adults, typically presenting with painless lymphadenopathy and a better prognosis. Radiologically, LCH is associated with bone damage, while RDD primarily presents with lymphadenopathy.
2. Plasma Cell Granuloma: Plasma cell granuloma is characterized by an inflammatory infiltrate predominantly composed of plasma cells, often accompanied by vascular proliferation and tissue necrosis (24). In contrast, RDD is marked by proliferation of histiocytes with large, pale-staining cytoplasm and the characteristic “infiltration” phenomenon, where histiocytes engulf lymphocytes. Immunohistochemically, RDD histiocytes are positive for CD68 (KP-1) and S100, while plasma cell granulomas show a characteristic immune phenotype with CD138 positivity (25). These pathological and immunohistochemical features allow for effective differentiation.
3. Erdheim-Chester Disease (ECD): ECD is characterized by foamy macrophages with a spindle shape, often with systemic involvement such as bones, heart, kidneys, and the nervous system. Radiologically, ECD typically presents with bilateral sclerosis or bone proliferation in long bones. Immunohistochemically, ECD is positive for S100 and CD68 but negative for CD1a, and it is commonly associated with BRAF gene mutations (26, 27). RDD, however, is mostly composed of macrophages that engulf lymphocytes, and immunohistochemistry shows positivity for S100 and CD68 but is negative for CD1a. RDD lesions typically involve painless lymphadenopathy in the neck, axilla, and groin, and the disease course is self-limiting with a good prognosis. ECD, on the other hand, often involves multiple organs with a poor prognosis. These distinguishing features help differentiate the two.
4. Meningioma: Meningiomas and RDD may overlap in clinical presentation and radiological findings, but they differ significantly in pathological and histological features. Meningioma is a common intracranial tumor that typically presents with symptoms such as headache, seizures, or neurological deficits (28). Radiologically, meningiomas are typically located in the dura or subdura, with CT or MRI showing enhancing masses with a clear boundary from the brain parenchyma. Pathologically, meningiomas are composed of lobulated tumor cells, often arranged in a “whorled” pattern, and immunohistochemically positive for EMA and Vimentin, but negative for S100. These pathological and radiological differences allow for the effective distinction between RDD and meningiomas (29).
5. Meningeal Lymphoma: Primary or secondary CNS lymphoma (meningeal lymphoma) is primarily composed of immature B or T lymphocytes (30, 31). Pathologically, meningeal lymphoma shows diffuse lymphocyte proliferation, and immunohistochemical staining is positive for CD20 (B-cell marker) or CD3 (T-cell marker), while S100 is negative (32). Radiologically, RDD often presents as a clear-marginated meningeal lesion with uneven enhancement, whereas meningeal lymphoma is typically a uniformly enhanced mass with adjacent brain edema and mass effect (31). The pathological and immunophenotypic differences can help differentiate the two and guide subsequent treatment.
6. Idiopathic Hypertrophic Pachymeningitis: Idiopathic hypertrophic pachymeningitis is a chronic inflammatory disease of the dura mater of unknown etiology, primarily presenting with diffuse thickening of the dura. Radiologically, it shows significant meningeal enhancement and is often accompanied by symptoms like headache and cranial nerve palsy (33, 34). Pathologically, idiopathic hypertrophic pachymeningitis shows fibrosis and chronic inflammatory cell infiltration without the “infiltration” phenomenon seen in RDD, and its immunohistochemical markers are nonspecific (35). In contrast, RDD lesions are usually localized and exhibit characteristic cytological changes, whereas hypertrophic pachymeningitis tends to present with widespread thickening and nonspecific inflammatory responses. These features can help distinguish the two.
7. IgG4-related disease (IgG4-RD): IgG4-RD is a fibroinflammatory condition characterized by tumefactive lesions, dense lymphoplasmacytic infiltration, storiform fibrosis, and an increased number of IgG4-positive plasma cells (36, 37). Given its ability to mimic RDD, particularly in cases involving pachymeningitis, careful differentiation is critical, especially in regions where IgG4-RD is more prevalent, such as China (38). IgG4-RD often involves systemic organs (e.g., pancreas, salivary glands) and exhibits storiform fibrosis, obliterative phlebitis, and abundant IgG4-positive plasma cells histologically. Elevated serum IgG4 levels and an IgG4/IgG ratio > 40% further support the diagnosis (38). In contrast, RDD is marked by histiocytes with lymphocyte emperipolesis, positive staining for S-100, CD68, and CD163, and lacks significant fibrosis or IgG4 expression. While IgG4-RD responds well to corticosteroids, RDD often requires surgical resection for localized lesions.

### 3.5 Treatment

As a rare neoplastic disease, RDD typically follows a self-limited course in most cases. However, a subset of patients may develop locally invasive or disseminated disease, leading to a poor prognosis (39–41). Currently, there is no internationally standardized treatment protocol for RDD, and treatment strategies primarily depend on the extent of organ involvement and the severity of clinical symptoms. The NCCN Clinical Practice Guidelines in Oncology: Histiocytic Disorders (2021) provides a reference framework for the diagnosis and treatment of RDD.

For localized lesions, surgical resection is both a diagnostic tool and the main treatment method (42, 43). Particularly in cases with isolated lesions, surgical excision can often lead to long-term remission or even cure. However, for multifocal or unresectable lesions, current treatment options include steroid therapy, immunosuppressants, chemotherapy, targeted therapies, and other adjuvant treatments (44, 45).

Steroid therapy is the most commonly used treatment method, with low-dose prednisone (1 mg/kg) or high-dose corticosteroids recommended, especially in cases involving vital organs or accompanied by systemic symptoms (46). Although steroid therapy often leads to significant symptom improvement, its long-term efficacy is limited, and there is a risk of steroid dependency (47). For patients with severe disease or those unresponsive to steroids, a combination of low-dose methotrexate with 6-mercaptopurine, cladribine, thalidomide, lenalidomide and other immunosuppressive agents and chemotherapy drugs may yield a better treatment response (20, 48, 49).

In cases with mutations in the MAPK/ERK signaling pathway, targeted therapies have shown high efficacy. For example, MEK inhibitors like Cobimetinib have been shown to significantly improve disease outcomes (50–52). Additionally, Rituximab, Sirolimus, and Chlorfarabine have shown promising results in some refractory cases (53). The application of genetic sequencing can be used to identify mutated pathways and guide personalized therapy, particularly in cases suspected to be familial RDD, where germline genetic testing is of great importance (9, 54, 55).

Surveillance and Intervention: For asymptomatic patients, a strategy of close follow-up is generally adopted. In contrast, patients with systemic symptoms or rapid progression require timely intervention to control disease progression. Personalized, multidisciplinary treatment strategies that take into account the patient’s disease characteristics, extent of involvement, and genetic features are essential for optimizing prognosis.

## 4 Discussion

Scalp-Cranial RDD is a rare form of histiocytic proliferation that can often mimic other intracranial lesions, such as meningiomas, due to its similar clinical and radiological features. This case serves as an important reminder that the accurate diagnosis of RDD requires careful evaluation of clinical presentation, imaging characteristics, and histopathological findings. The head CT and MRI findings in this case were consistent with the typical presentation of RDD, showing a localized lesion with characteristic high-density areas on CT and enhanced areas on MRI. This highlights the importance of imaging in preoperative diagnosis, as RDD often appears as a homogeneous mass on CT and shows isointensity or hyperintensity on MRI, with surrounding edema (56).

However, this case also underscores the potential pitfalls of relying solely on imaging. A more careful examination of subtle bone changes in the cranial plates, which were initially overlooked, could have led to a more comprehensive surgical approach. The failure to collect tissue from areas with abnormal bone structure during surgery demonstrated the importance of detailed preoperative imaging, especially for cranial bone abnormalities and subtle signal changes in bone structure that might not be immediately evident. This experience serves as a lesson for clinicians to focus on the fine details of imaging to avoid missed diagnoses and ensure a more complete surgical intervention.

Surgical resection of cranial RDD proved to be highly effective in this case. The patient underwent the successful excision of the affected dura mater and occluded superior sagittal sinus, with postoperative pathology confirming the diagnosis of RDD. Postoperatively, the patient showed no signs of recurrence during follow-up, which aligns with the generally favorable prognosis for localized RDD. Surgical excision remains the treatment of choice for isolated or localized lesions, especially in cases where complete resection is possible. This case provides strong evidence supporting the use of surgery as a safe and effective treatment for limited RDD in the CNS.

However, as this case demonstrates, multiple lesions or incomplete resections present a challenge, as there is currently no standardized treatment protocol for such cases. The role of systemic therapies, including steroids, immunosuppressive agents, and targeted treatments, has been discussed in the literature, particularly for patients with more widespread disease or those resistant to surgery (57). Recent studies suggest that targeted therapies, such as those directed at MAPK/ERK pathway mutations, may provide new therapeutic opportunities for refractory cases of RDD (58).

One of the common complications after cranial surgery, including those for RDD, is the development of subcutaneous fluid collections, which was observed in this patient. Subcutaneous fluid accumulation, while not uncommon, requires careful management, including aspiration and compression bandaging, as performed in this case, to prevent further complications. Although such collections are generally manageable, their occurrence underscores the importance of close postoperative monitoring and careful wound management to reduce the risks associated with postoperative recovery (59).

## 5 Conclusion

This case reinforces the importance of multidisciplinary collaboration in the diagnosis and treatment of RDD, particularly in rare and complex cases involving the central nervous system. Imaging, pathological evaluation, and clinical history must all be integrated to arrive at an accurate diagnosis, which can significantly affect the treatment approach and prognosis. In preoperative imaging, paying close attention to subtle changes in cranial bones and lesion characteristics can improve diagnostic accuracy and prevent surgical oversight.

While surgical resection remains the gold standard for localized RDD, more research is needed to establish optimal treatment strategies for multifocal or refractory cases. In these cases, an individualized treatment plan that incorporates genetic profiling, targeted therapy, and immunosuppressive treatments may provide better long-term outcomes.

This case offers valuable insight into the clinical management of scalp-cranial RDD, providing a foundation for future studies and enhancing our understanding of how to better diagnose and manage this rare disease in clinical practice. By considering personalized treatment strategies, including genetic testing and novel therapies, clinicians can optimize patient care and improve the overall prognosis for those with RDD.

## Data Availability

The original contributions presented in the study are included in the article/supplementary material, further inquiries can be directed to the corresponding author.
